# Supplementary material for: Enhancing Transdermal Delivery: Investigating the Impact of Permeation Promoters on Ibuprofen Release and Penetration from Medical Patches—In Vitro Research
Source: Int J Mol Sci. 2023 Oct 26;24(21):15632. doi: 10.3390/ijms242115632 (PMC10650847; doi:10.3390/ijms242115632)
Supplement: Supplementary file 1 [file ijms-24-15632-s001.zip › ijms-2659567-supplementary.pdf]

Supplementary Materials

Enhancing Transdermal Delivery: Investigating the Impact of Permeation Promoters on Ibuprofen Release and Penetration from Medical Patches—In Vitro Research

Paulina Bednarczyk [1], Anna Nowak [2], Wiktoria Duchnik [2], Łukasz Kucharski [2] and Paula Ossowicz-Rupniewska [1,*]

[1] Department of Chemical Organic Technology and Polymeric Materials, Faculty of Chemical Technology and Engineering, West Pomeranian University of Technology in Szczecin, Piastów Ave. 42, 71-065 Szczecin, Poland; bednarczyk.pb@gmail.com

[2] Department of Cosmetic and Pharmaceutical Chemistry, Pomeranian Medical University in Szczecin, Powstańców Wielkopolskich Ave. 72, 70-111 Szczecin, Poland; anowak@pum.edu.pl (A.N.); wiktoria.duchnik@pum.edu.pl (W.D.); lukasz.kucharski@pum.edu.pl (Ł.K.)

* Correspondence: possowicz@zut.edu.pl; Tel.: +48-449-4801

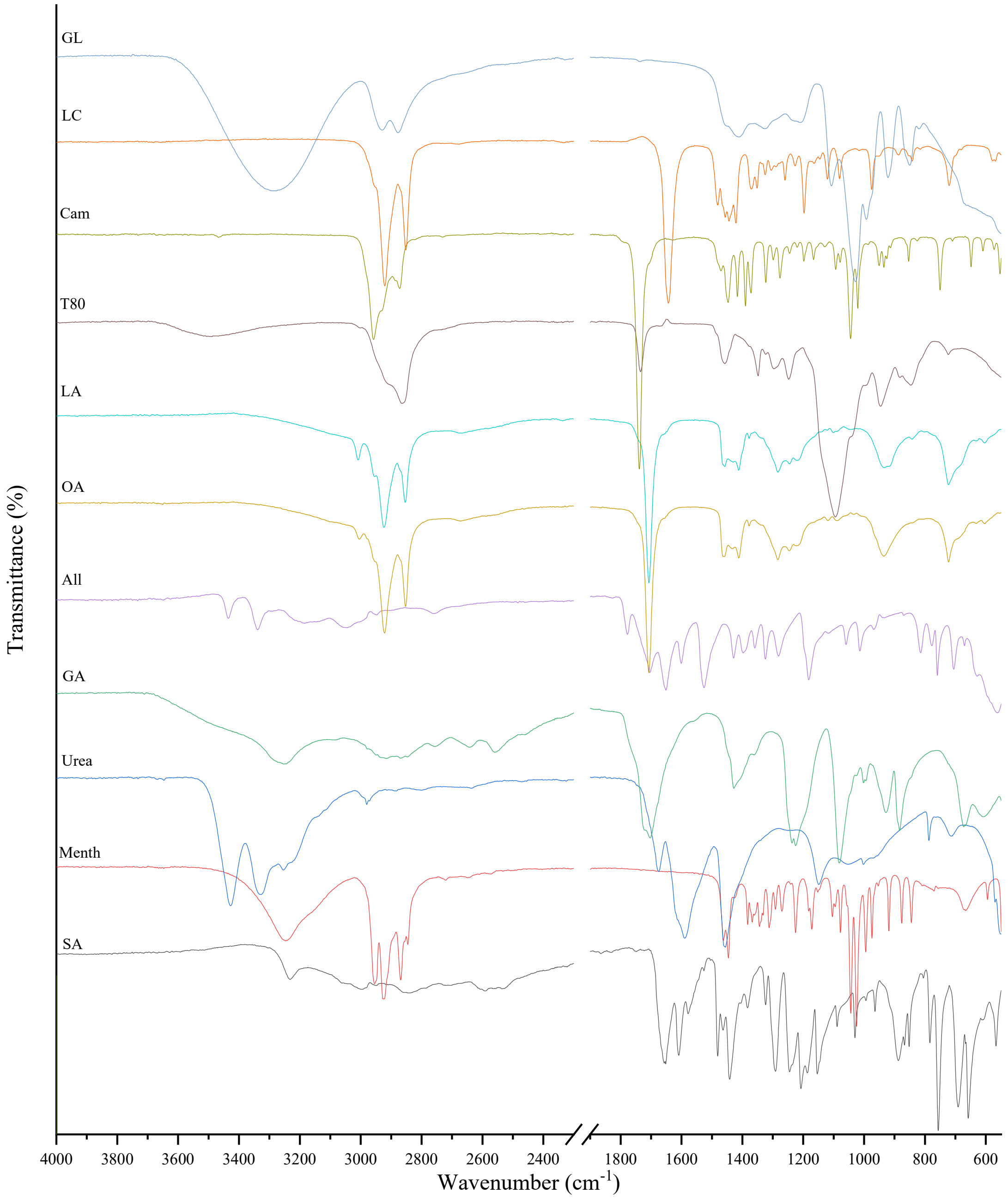

Figure S1. The FT-IR spectra of the pure form of the permeation promoters.

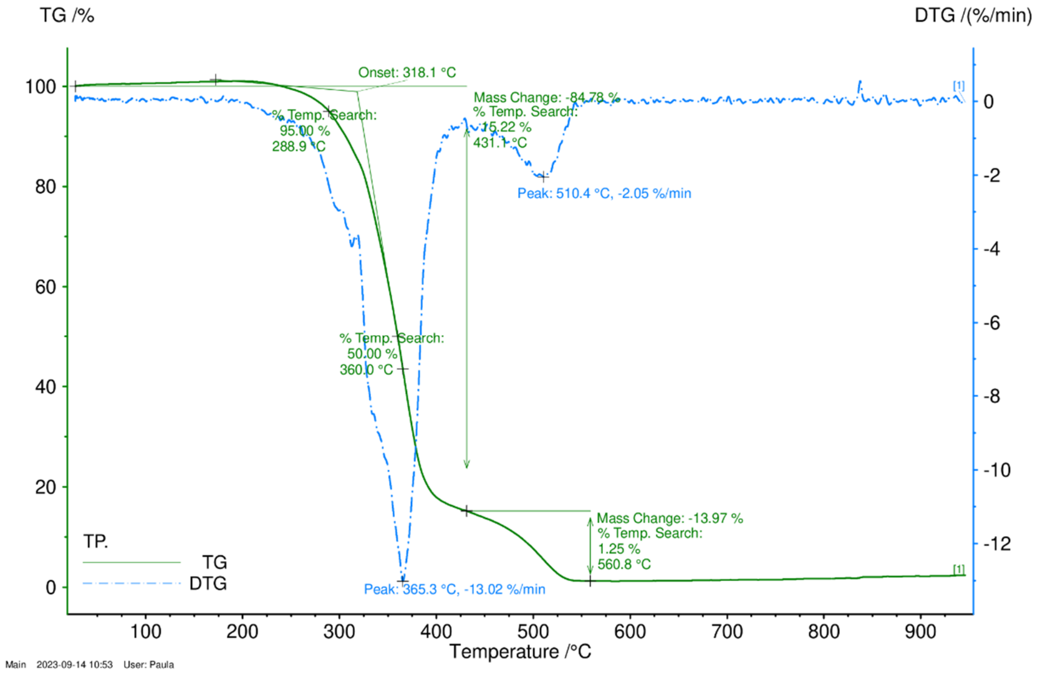

Figure S2. The TG and DTG curves of TP patch.

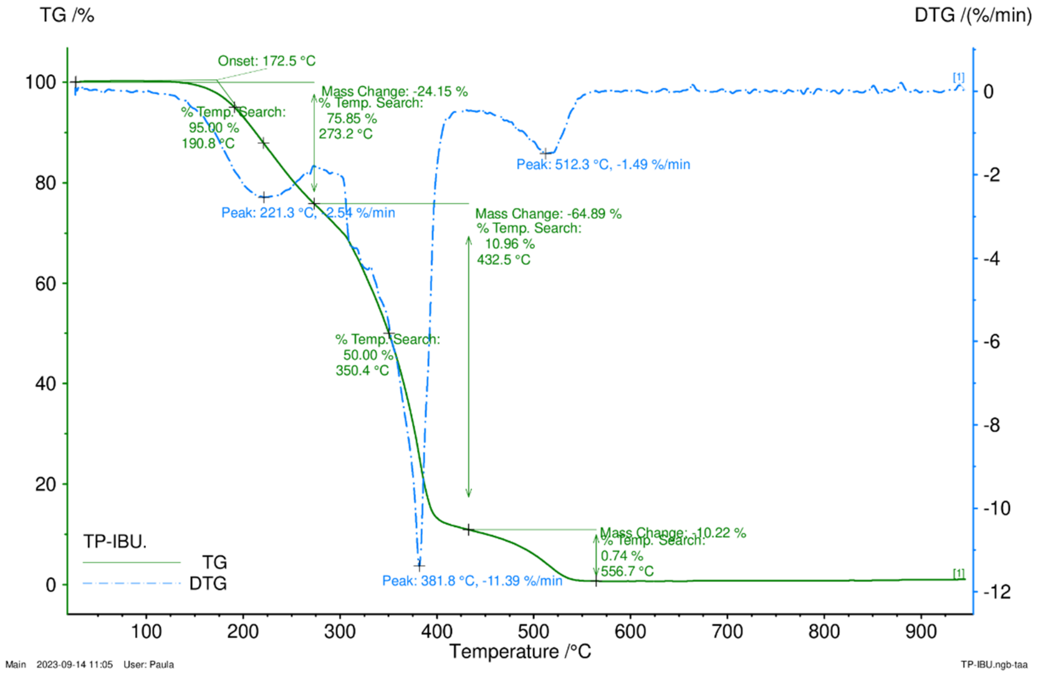

Figure S3. The TG and DTG curves of TP-IBU patch.

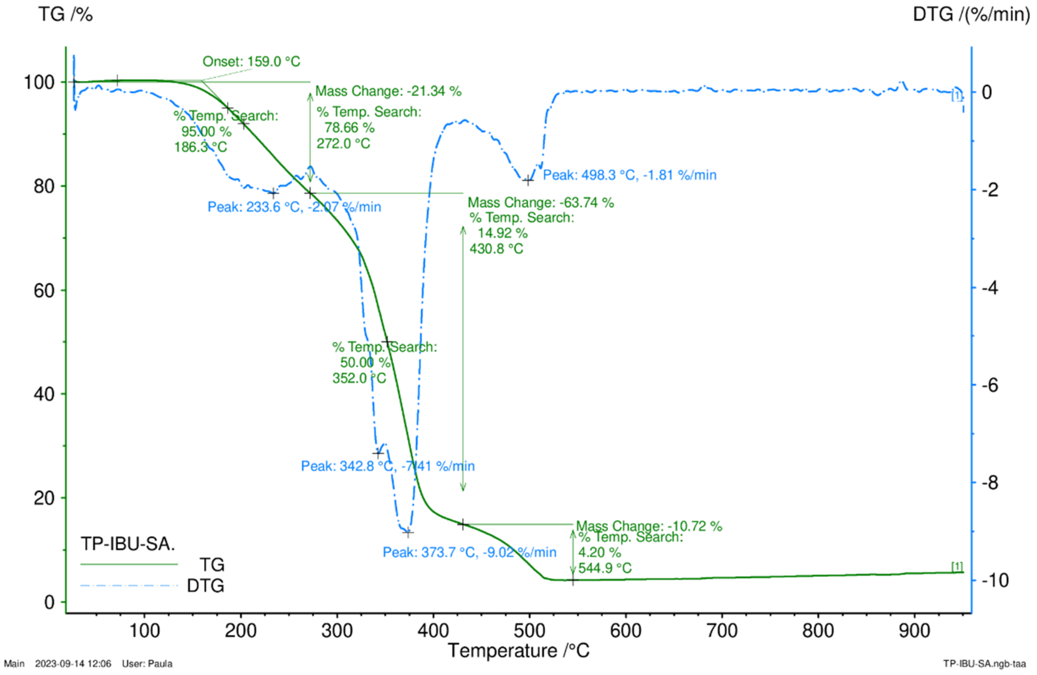

Figure S4. The TG and DTG curves of TP-IBU-SA patch.

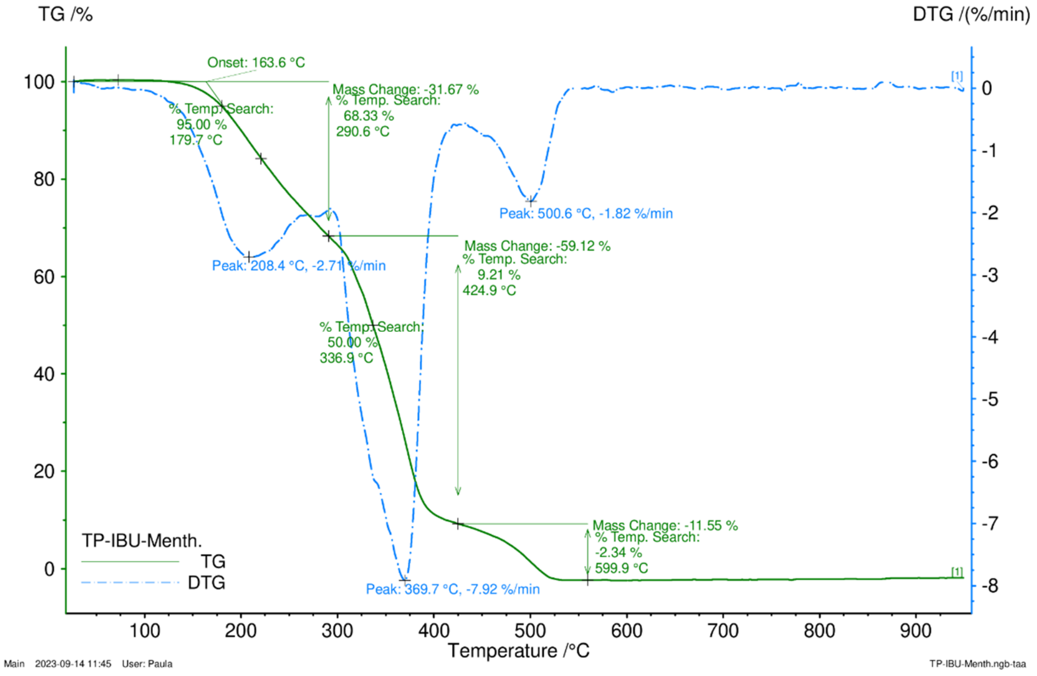

Figure S5. The TG and DTG curves of TP-IBU-Menth patch.

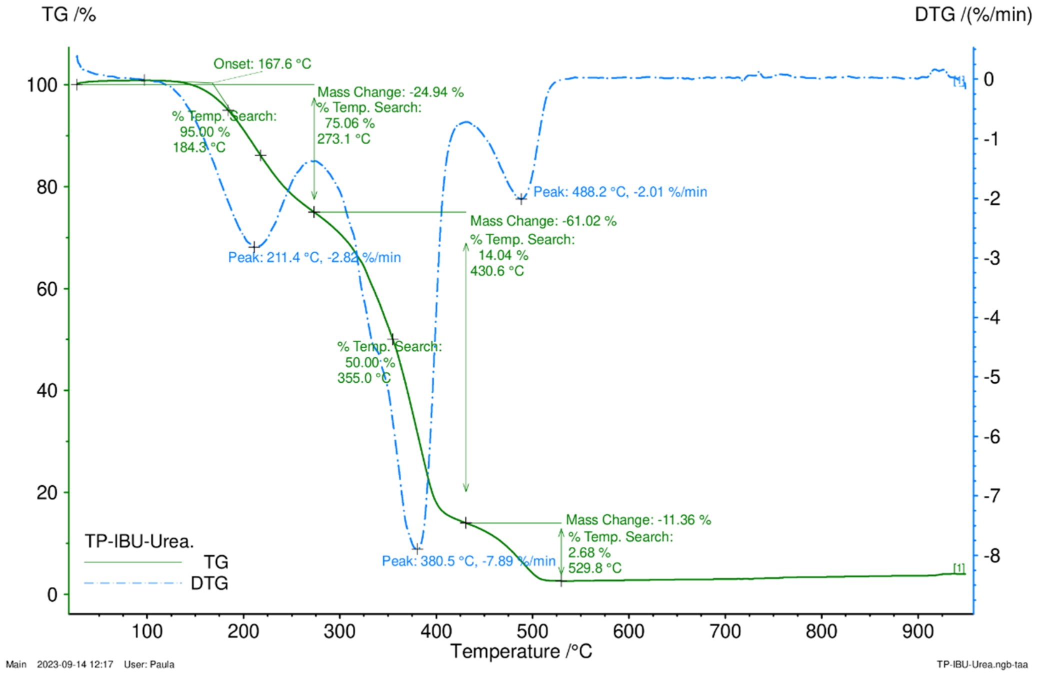

Figure S6. The TG and DTG curves of TP-IBU-Urea patch.

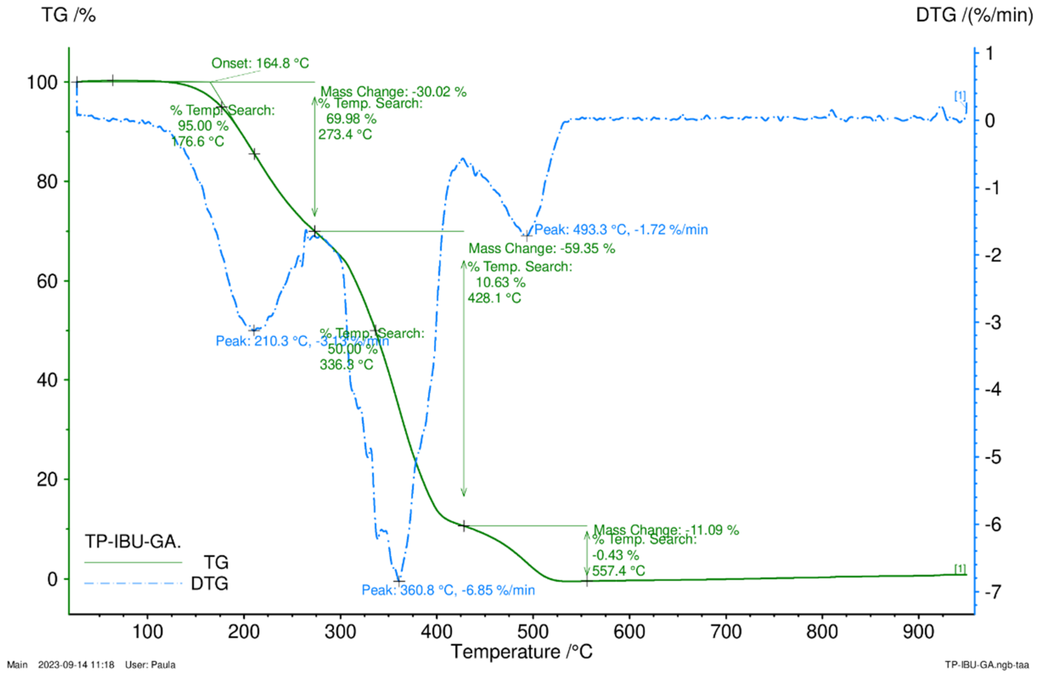

Figure S7. The TG and DTG curves of TP-IBU-GA patch.

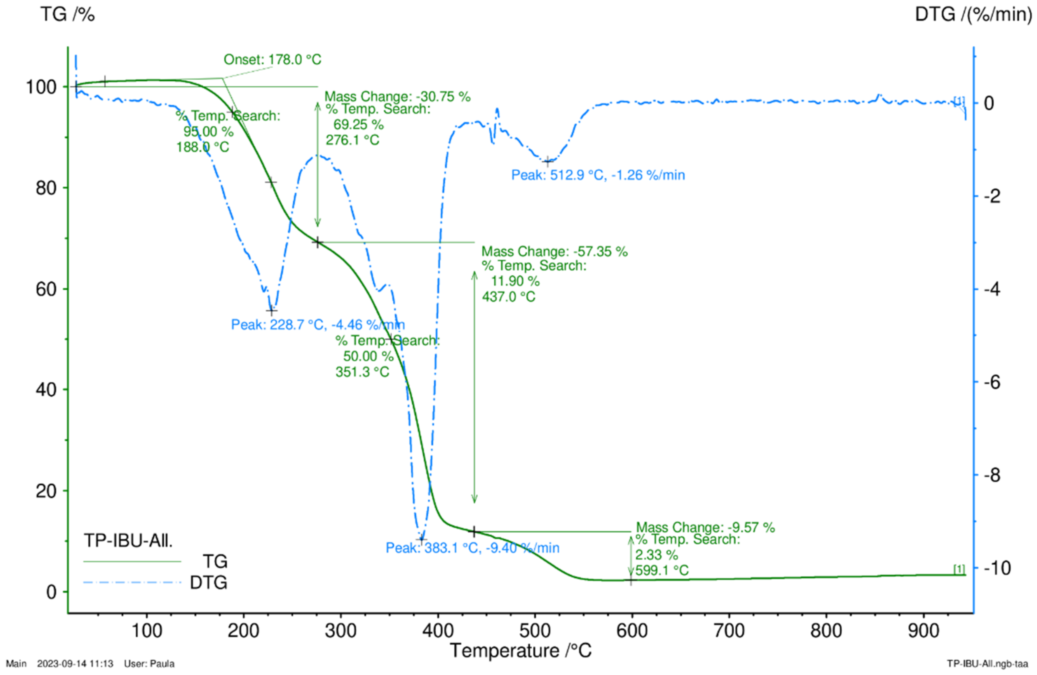

Figure S8. The TG and DTG curves of TP-IBU-All patch.

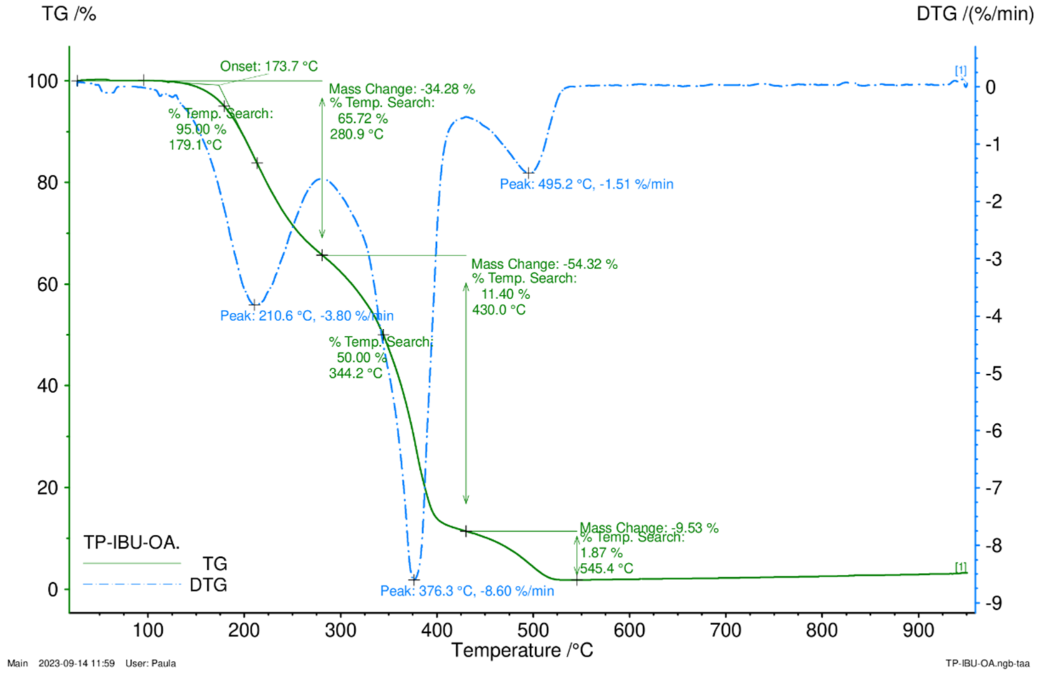

Figure S9. The TG and DTG curves of TP-IBU-OA patch.

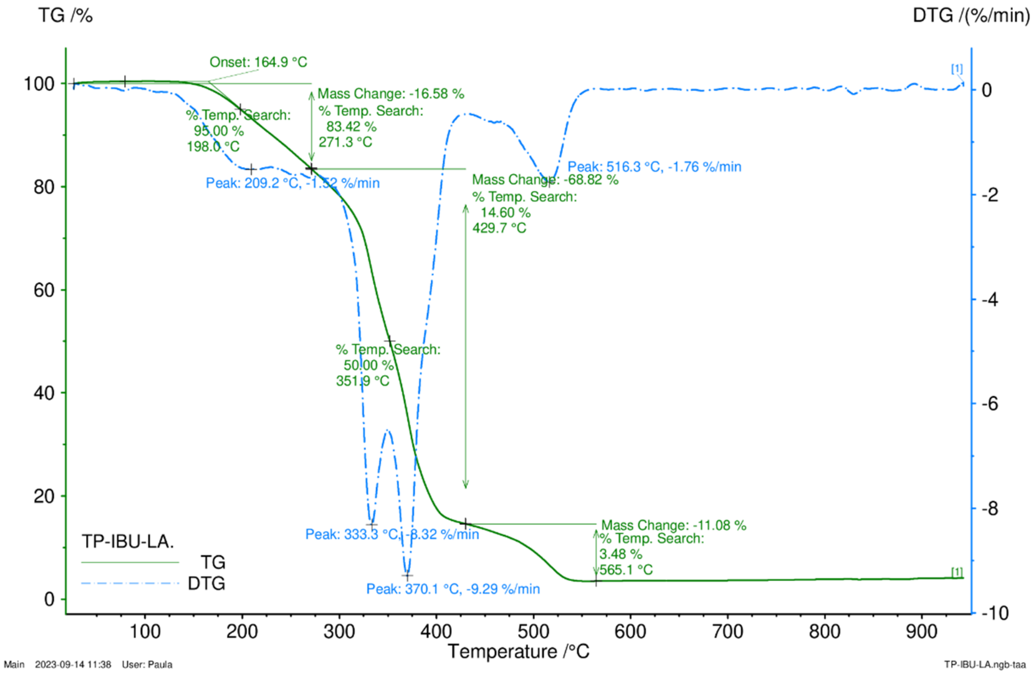

Figure S10. The TG and DTG curves of TP-IBU-LA patch.

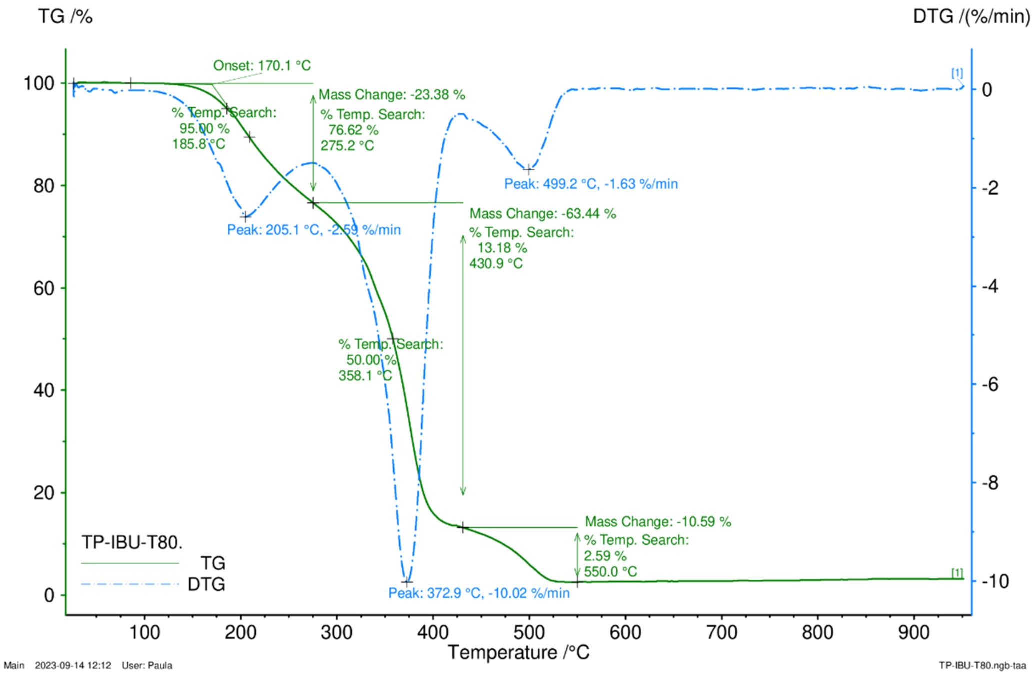

Figure S11. The TG and DTG curves of TP-IBU-T80 patch.

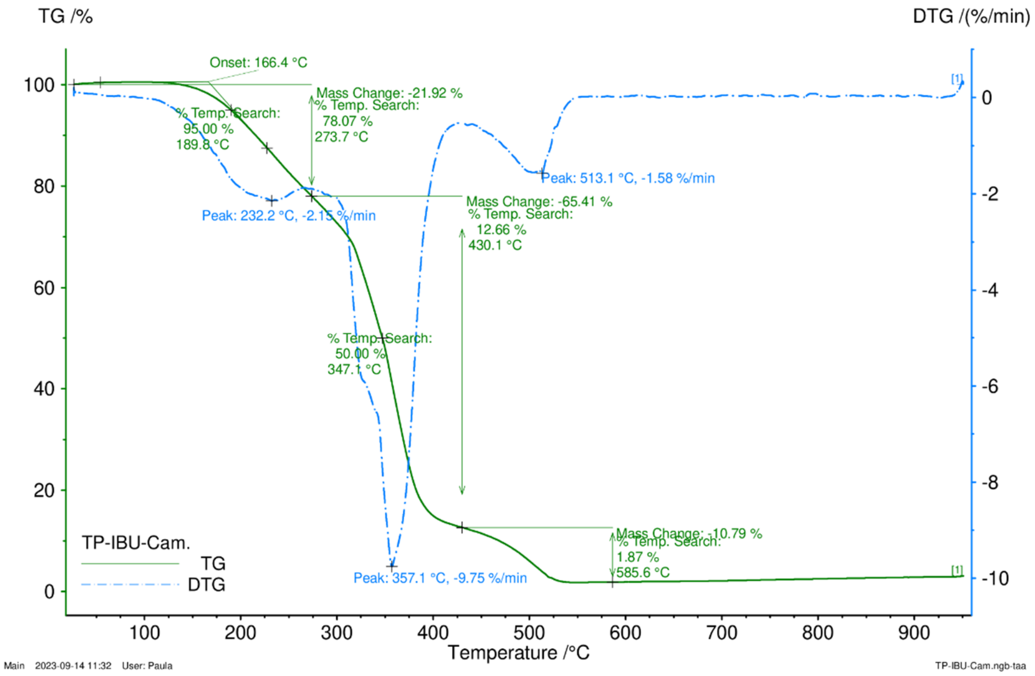

Figure S12. The TG and DTG curves of TP-IBU-Cam patch.

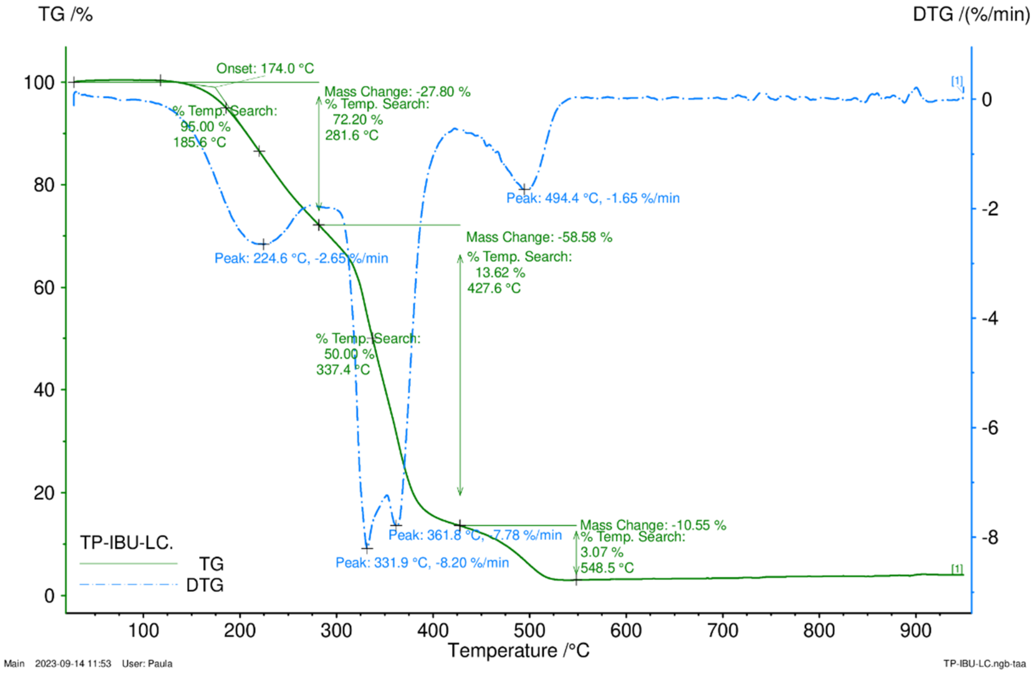

Figure S13. The TG and DTG curves of TP-IBU-LC patch.

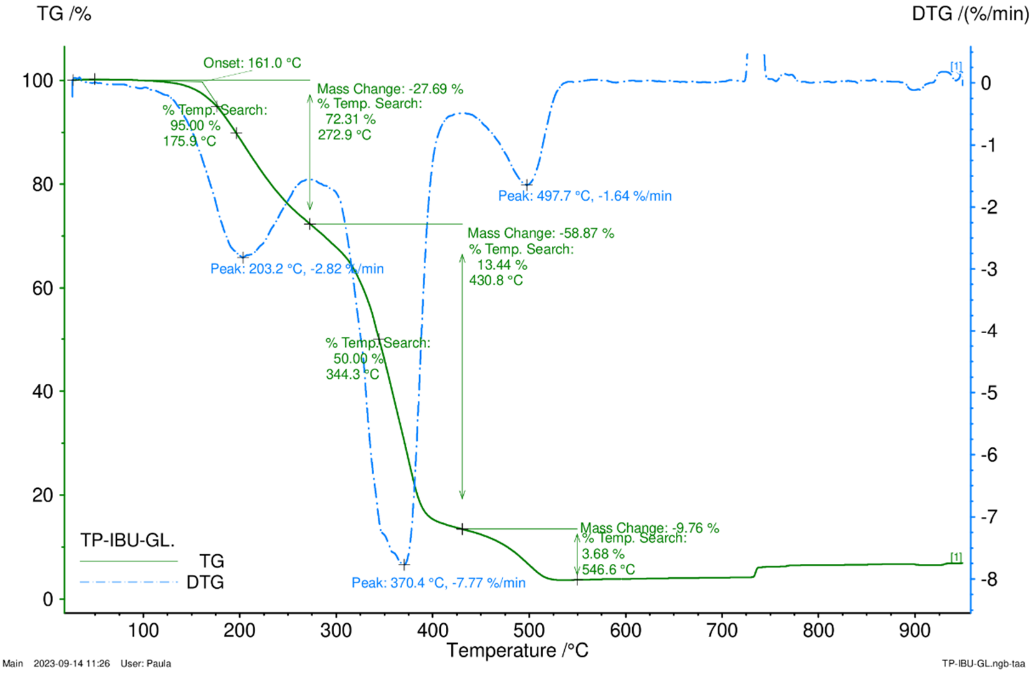

Figure S14. The TG and DTG curves of TP-IBU-GL patch.

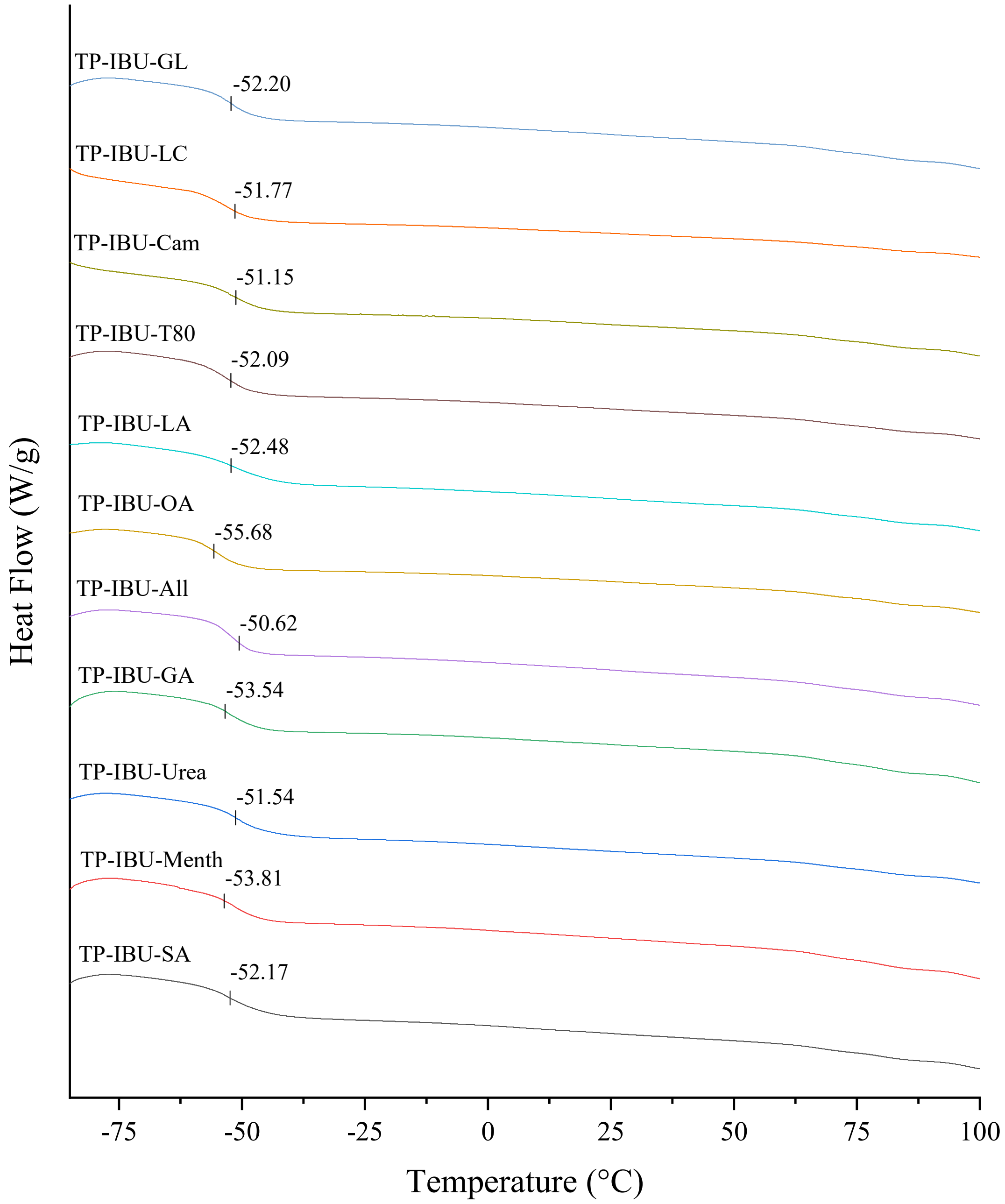

Figure S15. The DSC thermograms of TP-IBU patches with various permeation promoters.

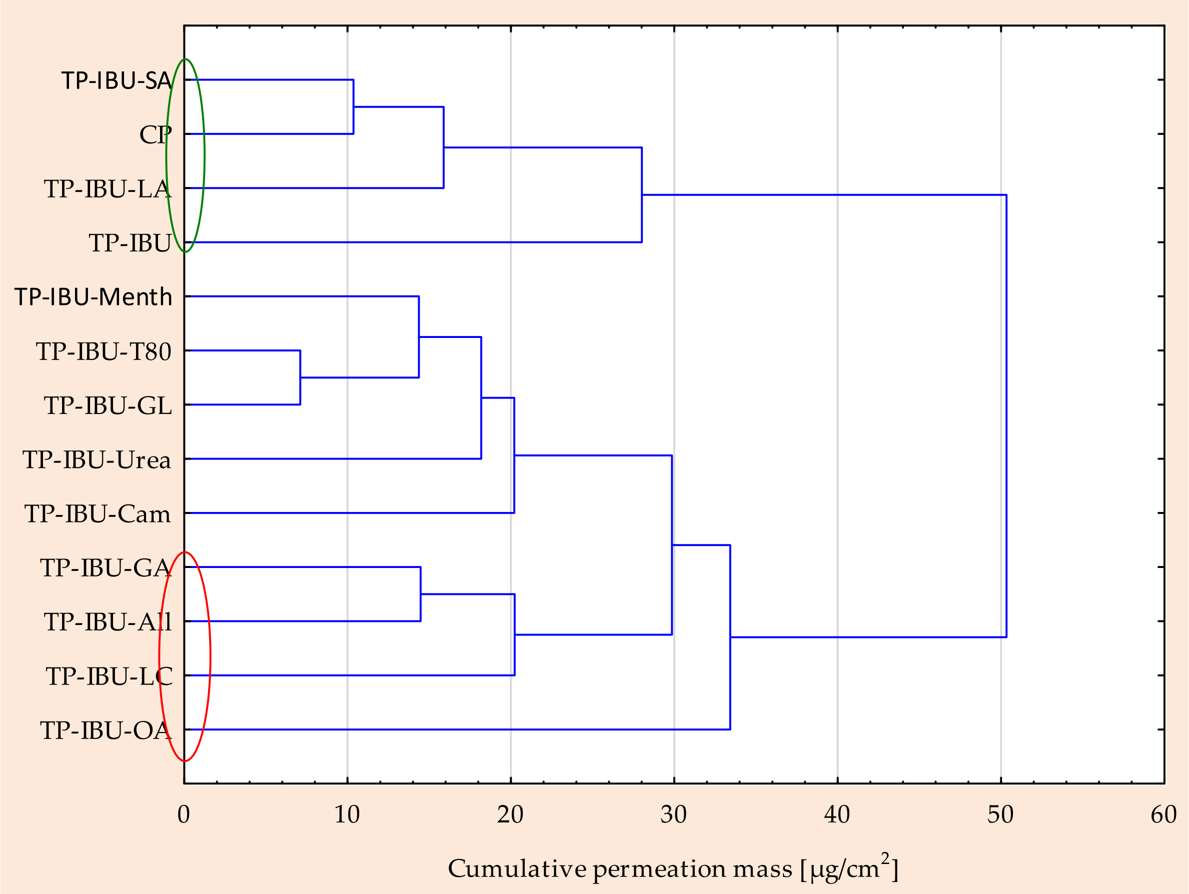

Figure S16. Cluster analysis graph for the mean accumulated mass of IBU after 24 h permeation through pig skin. The compounds form separate groups with similar permeability: the green circle is characterised by the lowest permeability, and the highest permeability characterises the red circle.

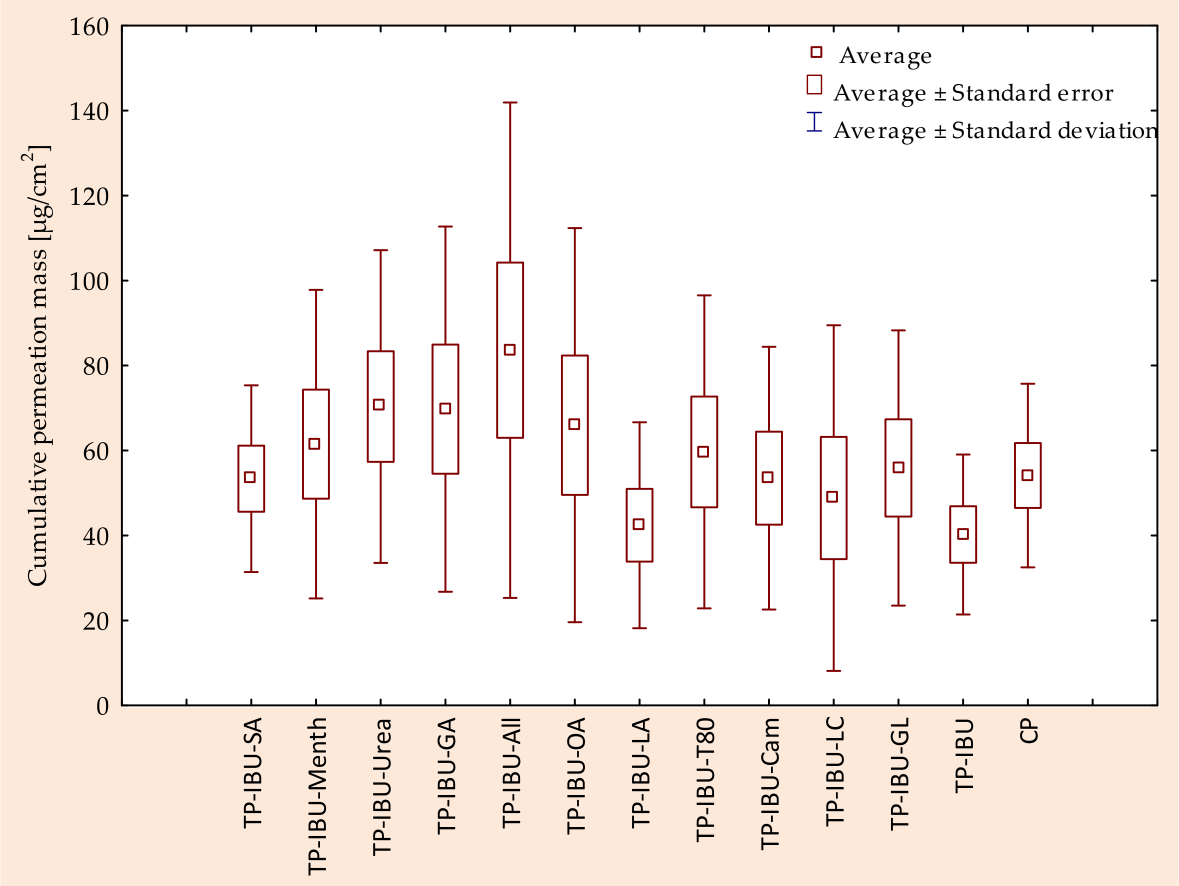

Figure S17. The box plot of cumulative mass of IBU throughout the entire 24-hour permeation.

Table S1. The cumulative mass of IBU after 24 hours of release *- significant differences of the derivatives compared to the control (patch without enhancer - TP-IBU) $p < 0.001$, $\alpha = 0.05$, mean ± SD, n = 3. The statistically significant difference was estimated by ANOVA using the Tuckey's test.

**Cumulative Release Mass [µg/cm²]**	
TP-IBU	683.260 ± 131.639
TP-IBU-SA	1151.911 ± 61.382 *
TP-IBU-Menth	1074.399 ± 66.544 *
TP-IBU-Urea	1220.918 ± 61.381 *
TP-IBU-GA	911.979 ± 36.937 *
TP-IBU-All	1218.487 ± 39.207 *
TP-IBU-OA	1309.038 ± 79.402 *
TP-IBU-LA	831.515 ± 65.425
TP-IBU-T80	990.641 ± 57.424 *
TP-IBU-Cam	979.647 ± 104.981 *
TP-IBU-LC	1126.922 ± 75.198 *
TP-IBU-GL	904.397 ± 74.915
CP	487.510 ± 68.442

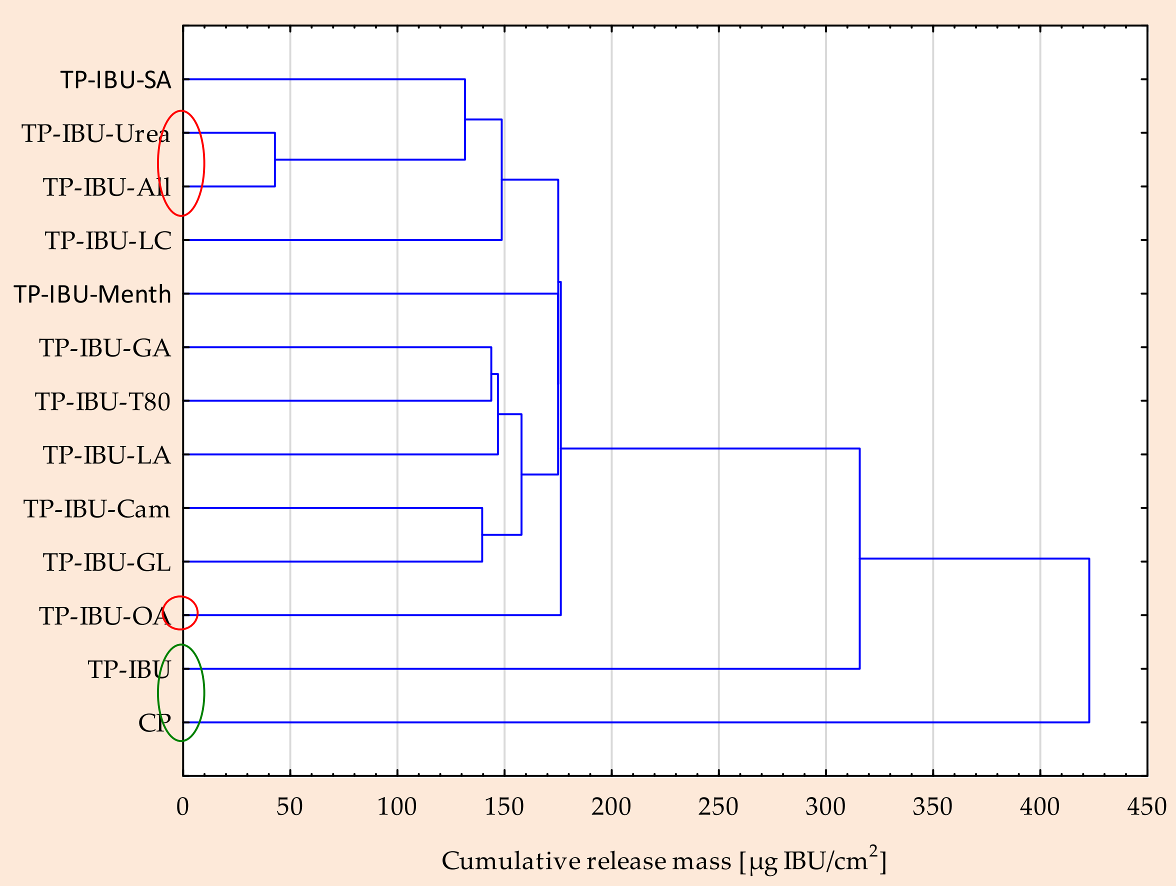

Figure S18. Cluster analysis graph for the mean accumulated mass of IBU after 24 h release. The compounds form separate groups characterised by similar releases; the lowest release characterises the green circle, and the highest release permeability characterises the red circle.

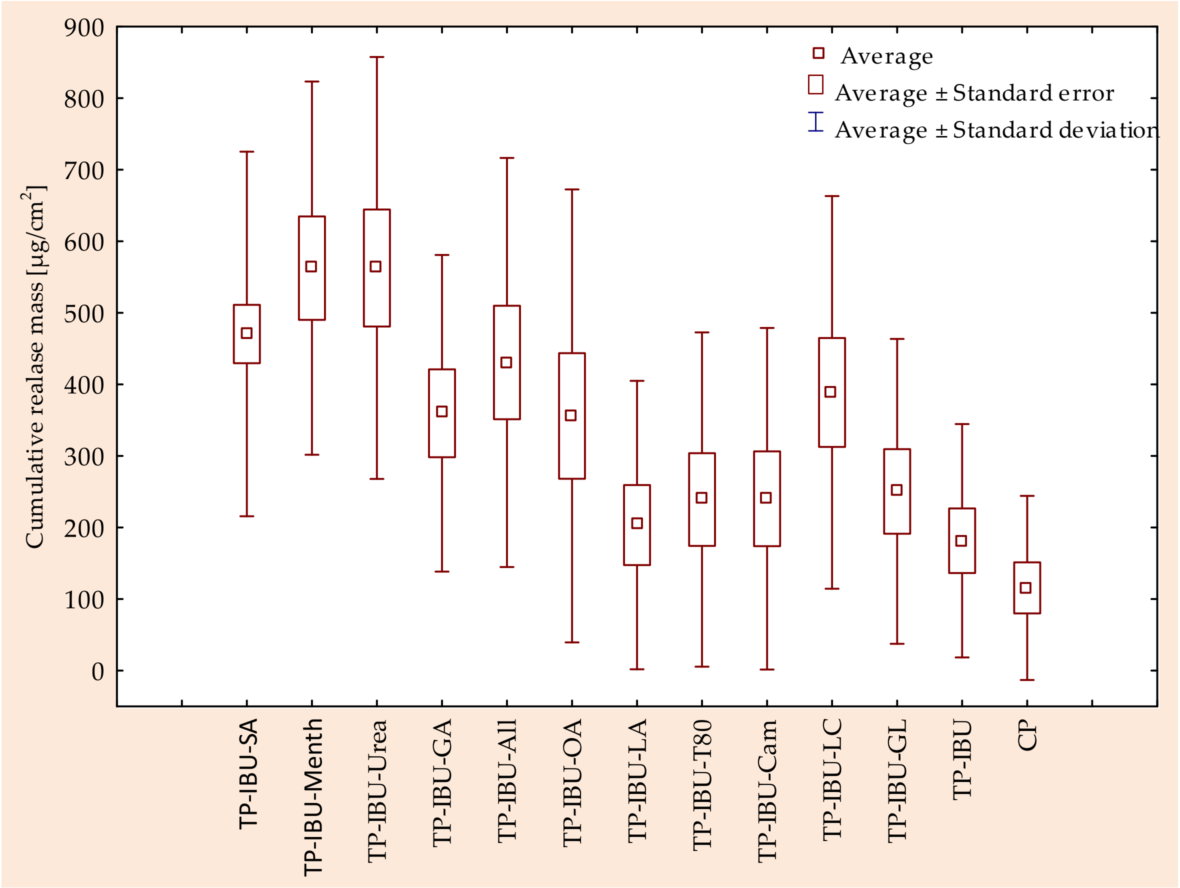

Figure S19. The box plot of cumulative mass of IBU throughout the entire 24-hour release.
